# RECODE - Relational Ecological COrpus for Data Extraction

**DOI:** 10.3897/BDJ.14.e177365

**Published:** 2026-04-17

**Authors:** Vasco V Branco, Lidia Pivovarova, Kari-E J Lintulaakso, Luís M Correia, Lenka Baranovičová, Iiris Lahin, Francisco Dias, David Filipe, Pedro Cardoso

**Affiliations:** 1 Finnish Museum of Natural History LUOMUS, University of Helsinki, Helsinki, Finland Finnish Museum of Natural History LUOMUS, University of Helsinki Helsinki Finland; 2 Centre for Ecology, Evolution and Environmental Changes (cE3c) & CHANGE - Global Change and Sustainability Institute, Faculdade de Ciências, Universidade de Lisboa, Lisbon, Portugal Centre for Ecology, Evolution and Environmental Changes (cE3c) & CHANGE - Global Change and Sustainability Institute, Faculdade de Ciências, Universidade de Lisboa Lisbon Portugal; 3 LASIGE and Departamento de Informática, Faculdade de Ciências, Universidade de Lisboa, Lisbon, Portugal LASIGE and Departamento de Informática, Faculdade de Ciências, Universidade de Lisboa Lisbon Portugal; 4 University of Helsinki, Helsinki, Finland University of Helsinki Helsinki Finland; 5 Department of Botany and Zoology, Faculty of Science, Masaryk University, Brno, Czech Republic Department of Botany and Zoology, Faculty of Science, Masaryk University Brno Czech Republic

**Keywords:** biodiversity, functional diversity, insects, large language models, machine learning, named entity recognition, natural language processing, occurrence data, species traits, spiders

## Abstract

**Background:**

Ecology, conservation biology and related disciplines are inherently data-based, with the success of many research projects and initiatives (e.g., protected areas, monitoring plans etc.) being directly dependent on the availability of location and trait information on species and populations. Unfortunately, this data is often either non-existent or available only as unstructured text within publications, especially for megadiverse taxa such as many invertebrate orders. With the emergence of large language models, there have been many attempts to automatically parse such data in machine-readable formats with variable success, either using prompt engineering or training models fit-for-purpose through named entity recognition and relation extraction. Model training has proven more efficient for complex data relations, but it needs labelled corpora, i.e. curated training data containing examples of this information for models to statistically learn from. This is a time-consuming process and, to our knowledge, no standard datasets exist upon which to train new and increasingly better models being released at an increasingly fast pace.

**New information:**

Here we describe RECODE, a manually annotated corpus of ecological and taxonomic literature, aimed at training and fine-tuning models for automated extraction of occurrence and trait data from unstructured text. All documents presented at this stage have been annotated and validated by experts familiar with the traits of the test taxa (spiders and insects).

## Introduction

Natural language processing (NLP) is the sub-field of machine learning that seeks to develop computer software capable of interpretation and replication of human language (Russel and Norvig 2021b). It includes a variety of methods, chief of which are large language models (LLMs). These are extremely high-dimensional neural networks, which, in turn, can be thought of as parallelisations of linear regression (Russel and Norvig 2021a). This high dimensionality is the result of training on a vast dictionary, allowing the LLM to process text in a probabilistic, non-conceptual way. This has made them particularly good at text analysis tasks. Two such tasks are named entity recognition (NER) and relation extraction (RE): placing tokens in an unstructured text in predefined categories and classifying the relationships between one or more tokens, respectively.

Within biology, NLP has been widely used, especially in molecular biology. A likely motivation is that, despite standardisation practices, a large amount of data in these fields is both complex and non-numeric. Researchers in the field routinely need to comb through large amounts of unstructured text in the form of patient records, scholarly publications and clinical narratives. This bottleneck attracted early collaborative work with data scientists and, as a result, information extraction (Nasar et al. 2018) from biomedical papers has been developing for more than twenty years (Blaschke (2002), Krallinger et al. (2008), Holzinger et al. (2014), Huang and Lu (2015), to name a few), leading to the creation of many high quality corpora suitable for NER and RE (Pyysalo et al. 2007, Vincze et al. 2008).

By contrast, use of NLP in conservation biology is still to take off in a similar way to molecular biology, mostly being restricted to tasks such as monitoring the trafficking of species on social media or analysing public consciousness on a topic. This is likely due to these tasks being simpler to model, requiring datasets that either are completely term non-specific, such as a simple two-column match between message and sentiment (e.g. Van Houtan et al. (2020), Acerbi et al. (2023)). These more common datasets do not discriminate between terms of interest or the textual relationships between them in a machine-interpretable way, making them unsuitable to train models for more complex tasks, such as data extraction. Additionally, datasets that do discriminate between ecological entities and their textual relationships may suffer from being overly generalist, not fitting specific use-cases. As such, more complex modelling such as LLMs has, by comparison, seen few recent applications (Abdelmageed et al. 2022), most being meta analyses to determine their reliability (e.g. prior knowledge, accuracy of information, consistent output structure etc.) of LLM in the field (Castro et al. 2024, Dorm et al. 2025).

In this paper, we seek to increase the degree of automation of NER in ecology and species conservation by presenting RECODE (Relational Ecological COrpus for Data Extraction), an annotation schema (i.e. an ontology or network of concepts and their relationships) and NER corpus of ecological terms. By establishing a fixed structure of terms in a machine-readable format and applying it consistently in documents (i.e. training data, our corpus), it allows computer software to deduce their place in documents through statistical correlation. Although the structure of RECODE is flexible enough to support variable project goals, it is primarily intended towards training and fine-tuning models capable of extracting data from scientific papers, as this data is currently "immobilised" in online literature repositories, inaccessible for ecology and conservation projects.

In RECODE, the central term is the binomial species name or another accepted taxonomic identifier (*Species*), the specific description of a place (*Loc*) and its coordinates (*Coord*), as well as the date at which it was found (*LocDate*). We intend it to be used for the extraction of trait names (*Trait*), their respective numerical measurements (*TraitVal*) and the metadata associated with each measurement. These pertain to both: 1) ecological aspects of the measurement: the sex of the specimen (*Sex*), their life stage (*LStage*) and number (*Count*) and 2) referential aspects: the coordinate format for latitude and longitude (*CoordSys*), the unit used to express a trait (*Unit*), the method for data aggregation or averaging as well as the variation or range (*Stat*), the original citation of the measurement (*Ref*), and the date on which it was recorded (*Date*). The result is a lightweight and hierarchical annotation framework that is easy to understand, making future additions to the corpus fast and low-cost.

Usage of RECODE also comes integrated in *R* package arete (Branco et al. 2025). Automated REtrieval from TExt (arete) is a pipeline and set of tools for the extraction of species data through LLMs. With it, users may easily convert RECODE data to specialised formats needed for machine learning (ML) services, such as .jsonl for GPT fine-tuning.

We provide all files in RECODE in the WebAnno TSV v.3.3 format. Additionally, the platform used in our annotation process, INCEpTION (Ubiquitous Knowledge Processing Lab 2024), allows for easy conversion from this format to several others (i.e CoNLL, NLP Interchange Format). All project data, both RECODE files themselves as well as the *R* scripts used for the figures in this paper, are publicly available on Zenodo, with the current working version of our guidelines being available on GitHub. Furthermore, this dataset is goal-oriented and published simultaneously with a complementary *R* package and a practical case of its usage for both fine-tuning and validation (Branco et al. 2024, Branco et al. 2025).

## General description

### Purpose

RECODE is a corpus of annotated scientific papers aimed at training and validating language models for extraction of occurrence and trait data from literature. Although right now it contains and has been tested only with spider and insect data, its ontology is not taxa specific and easily accommodates,and is intended, towards other taxa.

### Additional information

**Methodology**

**Data sourcing**

**Figure 1.**
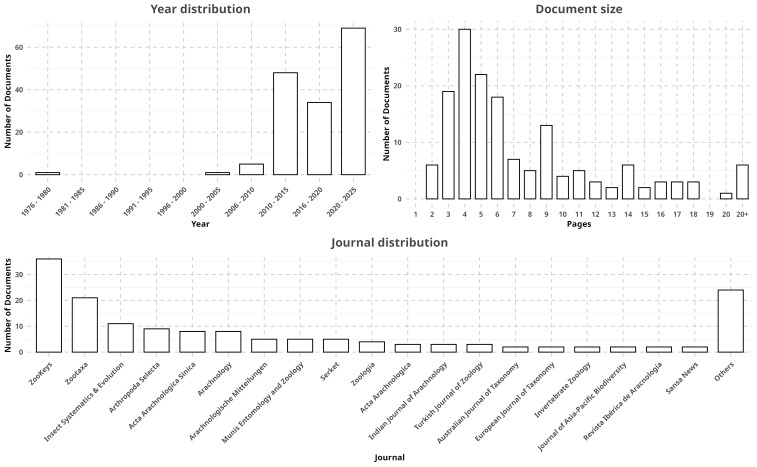
Metadata of documents in RECODE, reporting the number of documents: by year they were originally published (top, left); by the total amount of pages of each (top, right); by the journal in which they were published (bottom). Due to the high number of journals (n = 43), those with only one associated document were placed under "Others".

RECODE is an annotation schema and corpus of annotated papers, extracted from online literature repositories. For spiders, these have, for the most part, been obtained from those referenced and made available at the World Spider Catalog, an online reference and database for spider taxonomy (Natural History Museum Bern 2025). For insects, we used published material in known taxonomic journals. Although papers can vary quite a lot in size, we tended to process only papers under 20 pages in size. This was done for two reasons. First, we found large papers discouraged annotators, affecting speed and quality. Second, our annotation tool had document scalability issues: certain elements of the graphical user interface accumulated with each relation, severely cluttering the annotation space in long documents. The metadata for all RECODE entries can be consulted in its release in Zenodo (Fig. 1, see "Data resources").

**Named entity and relation selection**

**Figure 2.**
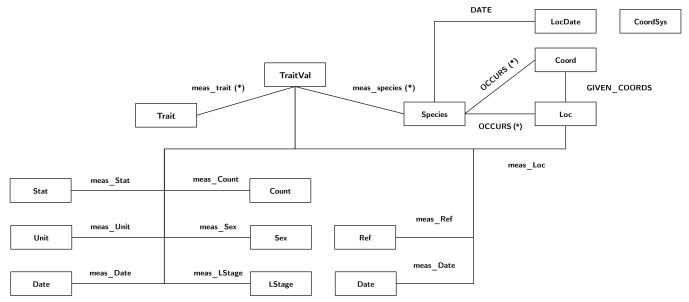
Annotation schema for named entities and relations in RECODE. Relations with an asterisk are mandatory for the starting named entities, i.e. trait values (*TraitVal*) may not be annotated by themselves, they must be connected to a trait name (*Trait*) and a species name (*Species*); species names may not be annotated by themselves, they must be connected to a set of coordinates (*Coord*) or a location (*Loc*).

The named entities (Table 1) and relations (Table 2) present in RECODE were determined from the observation of: 1) information available in taxonomic and ecological papers for spiders and insects and 2) information frequently missing in scientific literature that serves as major impediments to species knowledge and conservation. Of the information that we are missing, the two types of data that are most pressing to find an answer to are locality data and trait data (Cardoso et al. 2011). In order to tackle both and contribute to ease of access, two relation systems were created, one for occurrence data relations and one for trait data relations. The intersecting term for both systems is the binomial species name (*Species*). Additionally, the named entities are non-specific in nature, i.e. not dependent on species characteristics (e.g amount of wings etc). This leaves them general enough to be applicable to a wide variety of taxa in future versions (Fig. 2). Lastly, RECODE does not encode aggregate entities save exceptions (see Table 1, *TraitVal* and "Disentangling ambiguity" section).

**Disentangling ambiguity**

Ambiguity in defining named entities and their relations arises naturally from compromises meant to simplify the workload of the annotators. First, due to the complexity of the data being annotated, some categories were too wide in scope and needed to be restricted. As an example, we annotated as *Trait* only traits able to be numerically described, such as "chelicera length" or "number of eyes", meaning categorical data such as habitat type or food preference were not annotated at this stage.

Second, the complexity with which a concept is represented may vary due to differing degrees of standardisation. As an example, trait values (*TraitVal*) are an exception to a "one concept per entity" rule as they may contain both a single value or a spread of values and their summarising metrics. As these tend to be fixed in structure, we chose to keep them under the same named entity to simplify the annotators' task. As an example of the same issue, some named entities can be written in context dependent forms which affect annotation consistency. This is common with *Traits* where occasionally full meaning cannot be taken from a single token, but rather has to be interpreted in context. In the sentence "Abdomen: length 5 cm, width 1 cm", width does not make sense as a trait unless interpreted as "Abdomen [...] width". In these cases, annotators are encouraged to annotate all parts as a *Trait* for interpretation downstream.

Third, some categories share their name with existing ecological concepts which, in some cases, may come with undesirable assumptions. Terms are always relative to the documents present in the corpus, meaning RECODE was not designed to be compatible with outside references. For example, species names (*Species*) are tagged as found in-text without consideration over taxonomic nomenclature changes after the document's publishing.

All of these are expanded upon in the guidelines supplied to the annotators, described below and available in full on GitHub.

**Document selection**

Documents selected for RECODE followed, in a relaxed sense, two rules: 1) it has to be a majority (75%) in English. This was done in order to not exclude shorter papers with a version of the abstract in a different language; 2) size under a soft limit of 20 pages, due to the issues already described above (see "Data sourcing"). Validation checks with *arete* confirm that, although the size of the papers impacts the performance of the model, this impact is non-linear. The best performance is obtained when the entirety of the paper can be fit inside a model's context window, otherwise there is a drop in performance. However, after that, there is no difference between a document having to be separated twofold or twentyfold.

**Annotation guidelines and participant guidance**

The annotation process took place entirely in INCEpTION (Ubiquitous Knowledge Processing Lab 2024), an annotation tool developed and hosted by the Technical University of Darmstadt. The annotation phase of the paper took place after a short pilot phase, where the first author annotated papers 1 to 10, in order to determine early the feasibility of the annotation schema and considered workload. A total of six annotators contributed to RECODE. All annotators had previous experience working in life sciences and had an open line of communication with the experts supervising the annotation process. In order to further ensure a smooth process, all annotators had one short training session in which there was a joint annotation process with the first author in order to become familiarised with the process. Additionally, all annotators were supplied with a guidelines document developed during the pilot phase containing full text explanations for each named entity and for each relation, with examples and notes on potentially unintuitive cases. For example, we clarify that, for tagging "Count", the annotator should tag only the integer numbers of individuals associated with a given value and disregard the common mathematical notation of “n = ” for sample size. Annotators were encouraged to report on any inconsistencies of the guidelines and these can be consulted in full in Suppl. material 5.

**Annotator agreement**

**Figure 3.**
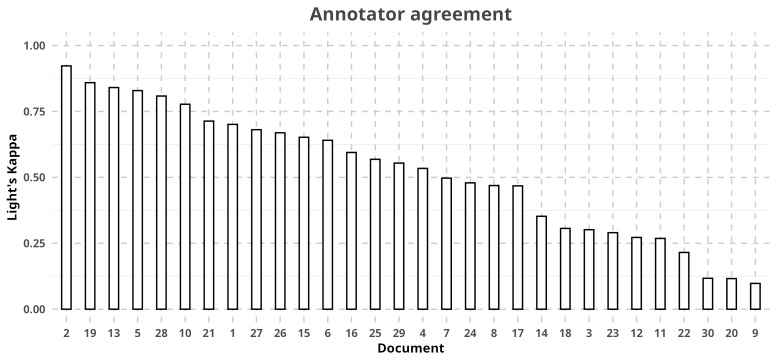
Annotator agreement score, expressed as Light's Kappa, for each of the 30 documents annotated for insects.

**Figure 4.**
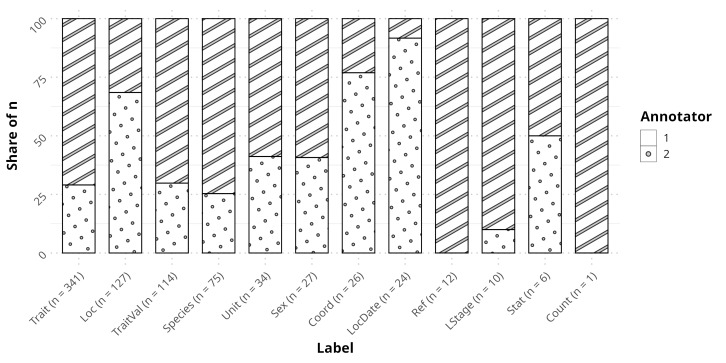
Stacked bar plot of annotator disagreement per named entity. For every named entity in the *x* axis, there is a total amount of disagreements, i.e. situations where, for a given token, one annotator annotated it as that named entity and the other did not. The share of that amount for each annotator is represented on the *y* axis. E.g: there were 341 situations where only one of the annotators labelled a token as a *Trait* and, in 71% of these instances, it was annotator 1 who labelled a token as a *Trait*.

A formal annotator agreement analysis was not calculated for documents describing spiders as annotators worked in different documents. This was due to a lack of resources at the time, prompting us to try to maximise the amount of documents annotated. Instead, documents present in RECODE were checked by experts before their final inclusion, with minimal disagreement between the annotators' work and that of the experts. However, on insect papers, we were able to allocate two annotators for the same set of papers and agreement could be calculated between them (Fig. 3). Originally, we expressed agreement with Light's Kappa (Light 1971), which indicates agreement between 0 and 1. Agreement was calculated to be near 1 with minimum deviance, indicating a perfect agreement (McHugh 2012). However, as this metric was not originally intended for relational data, a few elements were added: missing (NA) labels and relations were considered a category of their own, resulting in situations where one annotator labelling something and the other annotator not doing so was counted as a disagreement, along with any relations stemming from this label. This results in a metric that is much more conservative than the original version. In this modified Light's Kappa, annotators still had a mean agreement of 0.5198731 (0-1) and standard deviation of 0.2411671. To determine any sort of pattern to the disagreements between annotators, we performed an exploratory analysis on that subset of our data (Fig. 4). Specifically, we looked at differences in share, i.e. the number of times where an annotator marked a specific named entity and the other did not. We found that, although there were some labels with a high skew in annotator share (i.e. most times, this label is picked by the same annotator, e.g. *LStage*), these were mostly in those labels with few disagreements (annotator 1 share in six lowest: [mean, sd] = [61.90, 40.52]) and did not occur in those with many disagreements (annotator 1 share in six highest: [mean, sd] = [60.89, 15.80]). This suggests that annotator 1 was more active, but not necessarily biased towards specific labels and variability might be spurious. This is further supported by a Pearson's chi-square test between both sets of disagreements, which found them to be significantly related (X-squared = 126.71, df = 11, p-value < 2.2e-16). Additionally, while expected as the most populated label, *Traits* is the label most in disagreement and a revision of the guidelines might still be in order in a following version of RECODE. *Traits*, despite the streamlining already present in RECODE: 1) may still vary in ways unaccounted for in the guidelines (see "Disentangling ambiguity" for those already covered); 2) depend more on the annotators familiarity with the focus organism. Regardless and in practice for the user, agreement has been ideal and we would recommend in such cases to default to selecting the annotator with higher engagement.

## Geographic coverage

### Description

Global.

## Traits coverage

Traits that can be numerically described such as body part counts and measurements of length, width, weight etc.

## Usage licence

### Usage licence

Open Data Commons Attribution License

## Data resources

### Data package title

RECODE: Relational Ecological COrpus for Data Extraction

### Resource link

doi.org/10.5281/zenodo.15254437

### Number of data sets

1

### Data set 1.

#### Data set name

RECODE: Relational Ecological COrpus for Data Extraction

#### Data format

WebAnno TSV v3.3

#### Description

166 documents containing annotation data for papers and associated metadata.

## Additional information

### Results

**Named entities and relations**

**Figure 5.**
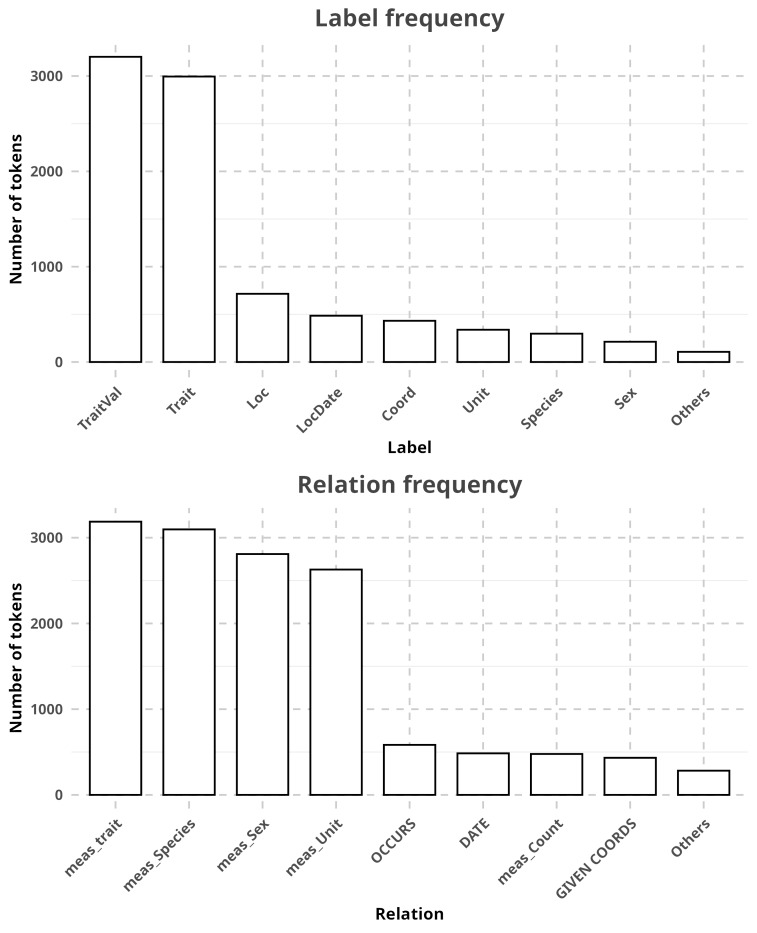
Frequency of named entities (i.e. labels, top) and relations (bottom) in RECODE. For visibility and due to the high amount of types of named entities (n = 14) and relations (n = 13) in our annotation schema, all categories representing less than 2% of the total amount have been grouped together under "Others". For relations, these were *meas_LStage*, *meas_Stat* and *meas_Ref*.

In RECODE, one document usually contains many instances of occurrence and trait data that, while making it more difficult to annotate, in the end, provides much more data for training. Most entities (78.66%) annotated were trait values (*TraitVal*, n = 3202, 36.43%), followed by traits (*Trait*, n = 2995, 34.07%) and localities (*Loc*, n = 716, 8.146%) (Fig. 5, Suppl. material 3). As for relations, the vast majority (84.15%) were *meas_Trait* (n = 3187, 22.78%), *meas_Species* (n = 3097, 22.14%), *meas_Sex* (n = 2810, 20.09%) and *meas_Unit* (n = 2629, 18.79%) (Fig. 5, Suppl. material 4).

**Taxonomy**

**Figure 6.**
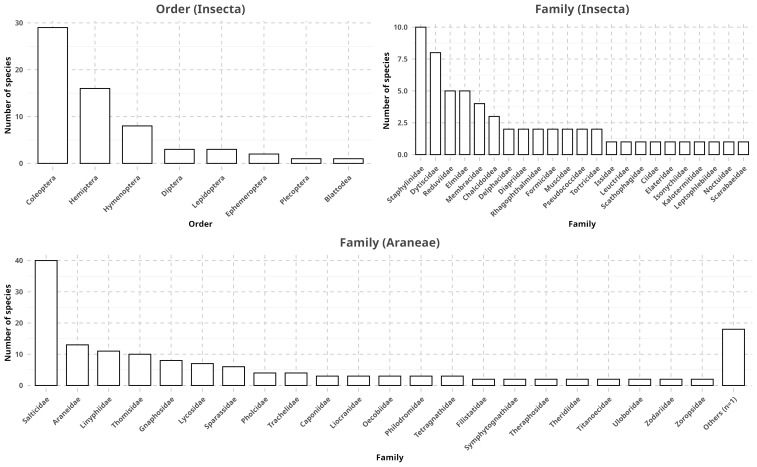
Taxonomic distribution of species mentioned in RECODE. Each species was counted only once per document. As insect documents had more than one annotator, the counts for each element were instead averaged between annotators. Due to the high number of spider families (n = 40), those with only a single representative were collected under "Others".

The three most populated (i.e number of species) spider families were Salticidae (n = 40, 26.31%), Araneidae (n = 13, 8.55%) and Linyphiidae (n = 11, 7.24%). At the genus level, we observe that Lyssomanes and Scopocira (each n = 4, 2.63%) and Caponina, Cuiambuca, Epeus, Lumptibiella, Oecobius, Wadicosa and Xysticus (each n = 3, 1.97%) were the most populated (Fig. 6, Suppl. material 1). This is in accordance with global data from the World Spider Catalog, in which Salticidae (n = 6809, 12.86%), Linyphiidae (n = 4941, 9.33%) and Araneidae (n = 3150, 5.95%) stand as the most species rich families (Natural History Museum Bern 2025).

As for insects, the five most populated orders were Coleoptera (n = 29, 46.03%), Hemiptera (n = 16, 25.40%), Hymenoptera (n = 8, 12.70%), Diptera (n = 3, 4.76%) and Lepidoptera (n = 3, 4.76%). Although not in order, this aligns with previous data from Stork (2018), which report Coleoptera (n = 386500, 38.1%), Lepidoptera (n = 157338, 15.6%), Diptera (n = 155477, 15.3%), Hymenoptera (n = 116861, 11.5%) and Hemiptera (n = 103590, 10.2%) as the five most populated orders globally.

At the family level, we observe that Staphylinidae (n = 10, 16.95%), Dystiscidae (n = 8, 13.56%) and Reduviidae (n = 5, 8.47%) were the three most populated. Finally, at the genus level, we observe that Allopachria (n = 7, 12.07%), Grouvellinus (n = 5, 8.62%) and Apatetica (n = 4, 6.90%) were the three most populated (Fig. 6, Suppl. material 2).

**Sources**

Despite a bias towards certain journals, namely Zookeys (n = 36, 22.92%), Zootaxa (n = 21, 13.37%) and Insect Systematics & Evolution (11, 7%), RECODE incorporates a diverse amount of sources (Fig. 1). Likewise, most of the sources used were originally published between 2020 and 2025 (n = 69, 43.67%), 2010 to 2015 (n = 48, 30.37%) and 2016 to 2020 (n = 34, 21.51%).

### Conclusions

The label and annotation composition of RECODE is consistent with what was expected. Trait values (*TraitVal*) are the largest category, which is expected considering it is one of the mandatory labels in our annotation schema. The near-absence of certain terms is also expected, such as outside references (*Ref*) as they are rarely used in taxonomical contexts. The same is true in relations. Some relations made between trait values and their characteristics obtain far larger counts. Sex is a good example as it is common for authors to describe a species in two sections, one for each sex, with the same named entity then being used in numerous relations (e.g. "Species example (male), Trait1 Value1, Trait2 Value2, Trait3 Value3, Trait4 Value4").

Our analysis of both the metadata and taxonomic distribution of data present in RECODE leads us to believe that we have both obtained a representative sample of taxonomic work in spiders and insects, as well as one that is convenient for annotators to work with. Some expected skews are present, such as in the publishing year and journal of sourced documents. In spiders alone, most papers describing species are recent, a trend matching the growing amount of new species described every year. Likewise, for journals, speciality taxonomic journals will always see more submissions of new species and within these, the most prestigious will take the lion's share.

**Limitations and Future work**

Due to resource limitations during the creation of our corpus, only insects could have multiple annotators working on each paper. In practical terms, we believe there was still a non-formal measure of agreement through the routine check-up and open line of communication between the annotators and experts, leading to continuous change in our guidelines. Regardless, a formal metric is yet to be developed. New annotations tools are also being considered in order to address our current scalability issues.

Additionally, some of the design choices in RECODE have important implications in its usage. Annotations are immutable, including biological terms that are subject to change such as species names (See "Disentangling ambiguity"). Any users seeking to perform downstream biological analyses must take care to independently relate annotated terms with any data.

There are several additions already planned out for RECODE. The first is the expansion of RECODE to include taxonomical descriptions of other taxa (e.g. crustaceans, mammals etc), giving priority to those groups described in vastly different ways to the papers already present. In this way, we would be covering the broadest scope of taxa. Another addition will be the inclusion of different versions of already present papers, generated with different OCR techniques. Although it is tempting to supply manually curated, "ideal" corpus elements, the reality is that, in order to be usable to the vast repositories of scientific literature available to us, a model must be able to disentangle common practical errors (loss of formatting, translation of special characters etc.) present in processing of documents through OCR. Additionally, although these errors may be disentangled through means other than machine learning, we believe it would still be invaluable, given it can scale up to process large numbers of papers. Finally, we plan to extend the corpus to ecology and other papers outside the current taxonomic scope, as these often present different structures. Due to the potential negative impact of novel structures on model performance, we encourage users interested in training models for taxa that are not currently covered in RECODE to carefully validate their results.

## Supplementary Material

AC0BD6CF-9762-57AD-8416-467BBC031704 10.3897/BDJ.14.e177365.suppl1 Supplementary material 1 Taxa table - Araneae Data type taxonomic Brief description A matrix containing every term annotated as a species, the original text they were annotated as, how many times that term was counted in that document, the ID of that document and the family and genus of that term, as well as any notes. File: oo_1450103.csv https://binary.pensoft.net/file/1450103 Vasco Branco

940AE6AA-C4E9-5D0A-8A9A-20722EBCF9ED 10.3897/BDJ.14.e177365.suppl2 Supplementary material 2 Taxa table - Insecta Data type taxonomic Brief description A matrix containing every term annotated as a species, the original text they were annotated as, how many times that term was counted in that document, the ID of that document and the order, family and genus of that term, as well as any notes. File: oo_1450104.csv https://binary.pensoft.net/file/1450104 Vasco Branco

18DEBB9E-D1D5-51F1-906F-3179685621AA 10.3897/BDJ.14.e177365.suppl3 Supplementary material 3 Count of labels in RECODE Data type metadata Brief description Every label present in RECODE, as well as their absolute and relative (percentage) counts. File: oo_1450083.csv https://binary.pensoft.net/file/1450083 Vasco Branco

1B8ACFE6-EE6C-5D22-8979-CFDFC41B18AB 10.3897/BDJ.14.e177365.suppl4 Supplementary material 4 Count of relations in RECODE Data type metadata Brief description Every relation present in RECODE, as well as their absolute and relative (percentage) counts. File: oo_1450084.csv https://binary.pensoft.net/file/1450084 Vasco Branco

ACC3D9EA-9F83-5411-9C53-432E46CC268A 10.3897/BDJ.14.e177365.suppl5 Supplementary material 5 Annotator guidelines Data type text Brief description Original guidelines document developed for the annotators with explanations on each label and relation, examples and notes on potentially unintuitive cases. File: oo_1392268.docx https://binary.pensoft.net/file/1392268 Vasco Branco

## Figures and Tables

**Table 1.** Named entities annotated in RECODE. Context examples in some cases might contain different named entities, with the one in focus being marked in bold.

NER Tags	Definition	Examples
Species	Original character string provided as species name or taxon identifier by the data provider.	"**Silometopus ambiguus** (figs. 10-12): BELGIUM: West-Vlaanderen: Knokke, Zwin Nature Reserve (51.367°N,3.367°E, 2 m a.s.l.), 2 males, 6 females, pitfall traps in salt marsh, 2.–16. Jun 2014, J. Van Keer leg., R. Bosmans coll."; “Pan paniscus”; “Arachnida”; “C. planirostris”; “L. brasiliense”
Loc	The specific description of a place.	"Silometopus ambiguus (figs. 10-12): **BELGIUM: West-Vlaanderen: Knokke, Zwin Nature Reserve** (51.367°N,3.367°E, 2 m a.s.l.), 2 males, 6 females, pitfall traps in salt marsh, 2–16 Jun 2014, J. Van Keer leg., R. Bosmans coll."; “Florida”; “Germany”
LocDate	The date at which a Species was observed in a specific location (Loc).	“Type material: Holotype: Male collected from banana agro-ecosystems near Raver (21.25 ° N, 76.03° E), Distt. Jalgaon, Maharashtra, India, **21- 25 December 2012**, Seema Keswani, deposited at Arachnology Museum, Forest Training Institute, Chikhaldara, Maharashtra-India”; “10.09.2009”; “3 March 2022”; “24.IV.2009”
Coord	The verbatim original spatial coordinates of a Location (Loc). The coordinate system when available should be tagged as the CoordSys.	"Silometopus ambiguus (figs. 10-12): BELGIUM: West-Vlaanderen: Knokke, Zwin Nature Reserve (**51.367°N,3.367°E**, 2 m a.s.l.), 2 males, 6 females, pitfall traps in salt marsh, 2–16 Jun 2014, J. Van Keer leg., R. Bosmans coll."; (39.8210N 2.7951E); (-41.0983, -121.1761); 17T 630084 4833438; 41 05 54S 121 05 34W; -77.508333, 164.754167; 77° 30.5' S, 164° 45.25' E; 77° 30' 29.9988" S, 164° 45' 15.0012"; E -1314485.732632, 358267.239976
CoordSys	The coordinate format or datum, for terms labelled as Coord.	"decimal degrees"; "degrees decimal minutes"; "degrees minutes seconds"; "UTM"; "EPSG:4326"; "WGS84"; "NAD27"; "Campo Inchauspe"; "European 1950"; "Clarke 1866"
Trait	Original character string provided as trait label by the data provider. This may include abbreviations of trait names.	“Tail length”; “greatest length of skull”; “GLS”
TraitVal	Measurement trait values. A trait value can only be a numerical measurement along with metrics describing the ranges of calculated data including minimum value, maximum value, mean value, standard deviation and number of specimens. In this case, the entirety of the measurement and all metrics must be labelled as a single entity.	"Description. Female (Type 1): Total length **4.85-5.29** (LG01-03, n = 3). One specimen (LG01) measured: body length **5.20**; cephalothorax **2.21** long, **2.17** wide; abdomen **2.99** long, **2.48** wide (Fig. 1)". 33.57 (31.36-36.56, 50); 29.5-34.6; 6.5, 7.5; 12.7 ± 0.2
Unit	The unit value of the measured trait. These values should only be tagged if they are describing the unit of the trait value measurement. Irrelevant data units should be avoided.	"g"; "mm"; "cm"; "kg"; "inches"; " 15 **in**"
Count	Number of specimens. It must be a numerical value either in number or textual form. It should be only tagged as mentioned separately in examples. If they are part of the range then do not tag them separately as this is part of the range now.	"Silometopus ambiguus (figs. 10-12): BELGIUM: West-Vlaanderen: Knokke, Zwin Nature Reserve (51.367°N,3.367°E, 2 m a.s.l.), **2** males, **6** females, pitfall traps in salt marsh, 2–16 Jun 2014, J. Van Keer leg., R. Bosmans coll."; "**15** males"; "**One** female, **three** of the specimens."; "**10** females."; "Specimen of **15** organisms"; "n = **16**"
Stat	The method for data aggregation or averaging as well as the variation or range.	"In Brazil (Vizotto and Taddei 1976), **means SD (mm; range, coefficient of variation)** for 15 males and 15 females, respectively."; "means (mm; ranges)"; "SD (mm; range, coefficient of variation)"
Sex	The sex of the biological individual(s). This should imply sexes whose trait data is given and can be either a noun or a sex symbol, ♂, ♀, ☿.	“male”; “female”; “unknown”; “hermaphrodite”; “♂♂”; “m”; “f”; “mf”; “mm”; “ff”
LStage	The age class or life stage of the biological individual(s).	“juvenile”; “juv.”; “j.”; “jj.”; “subadult”; “adult”; “ad.”; “egg”; “larva”; “pupa”; “spiderling”; “instar”; “nymph”
Ref	A citation that references the measurements of trait data. There can be many citations in the document, but only the ones that relate to providing trait information should be tagged.	"**Gimenez and Giannini (2016)** provided means (mm; ranges) of craniodental measurements."; "**Peters et al. (2002)** reported the following means (mm; ranges) for six females and 12 males, respectively"; "In Brazil (**Vizotto and Taddei 1976**) , means SD (mm; range, coefficient of variation) for 15 males and 15 females, respectively".
Date	The date on which a trait value was recorded.	“1900”; “April 1900”; “12 Feb 1900”; “12.02.1900”; “02-12-1900”

**Table 2.** Relational terms annotated in RECODE.

Relation	1st entity	2nd entity	Summary
**Occurrence data**			
OCCURS	Species	Loc / Coord	Occurrence of a species at any locality. If unpaired, i.e. described only with a set of coordinates. Coord is the end term, otherwise Loc takes precedence.
DATE	Species	Loc_Date	
GIVEN COORDS	Loc	Coord	If a location is described with both a Loc and Coord entities, this relation is established between them.
**Trait data**			
meas_Species	TraitVal	Species	Trait value of a species linked with its name.
meas_Trait	TraitVal	Trait	Trait value of a species linked with the name of said trait.
meas_Unit	TraitVal	Unit	Trait value of a species linked with the name of the unit used to express it.
meas_Stat	TraitVal	Stat	Trait value of a species linked with meta information on its elements.
meas_Loc	TraitVal	Loc	Trait value of a species is linked with the locality where the individual(s) with that trait were found.
meas_Count	TraitVal	Count	Trait value of a species is linked with the the count of individual(s) with that trait.
meas_Sex	TraitVal	Sex	Trait value of a species is linked with the sex of the individual(s) with that trait.
meas_LStage	TraitVal	LStage	Trait value of a species is linked with the life stage of the individual(s) with that trait.
meas_Ref	TraitVal	Ref	Trait value of a species is linked with the external reference used to support it.
meas_Date	TraitVal	Date	Trait value of a species is linked with the date where its measurement was taken
