# Supplementary material for: RECODE - Relational Ecological COrpus for Data Extraction
Source: Biodivers Data J. 2026 Apr 17;14:e177365. doi: 10.3897/BDJ.14.e177365 (PMC13109734; doi:10.3897/BDJ.14.e177365)
Supplement: Supplementary material 5 — Annotator guidelines [file bdj-14-e177365-s005.docx]

**ANNOTATION GUIDELINES WITH INCEPTION**

# 1. INTRODUCTION

First let’s have a brief introduction about the entities that are involved in tagging any scientific journal or publication for the purpose of identifying useful trait information about a species. The annotations are based on measurements of trait information of a species and its secondary information. These bits of information in every measurement can be classified in following categories:

- **ETS-Term** The Ecological Traitdata.
- **NER Tags** The corresponding Tag applied in the document.
- **IOB Tags** The generated tags according to Conll structure.

The ETS-fields are annotated using NER-Tags that are listed in Table1

**TABLE 1: ANNOTATION ENTITY TAGS AND DEFINITIONS**

| **ETS-Term** | **NER Tags** | **IOB Tags** | **Definition.**  **See:** [**https://terminologies.gfbio.org/terms/ets/pages/**](https://terminologies.gfbio.org/terms/ets/pages/) | **Examples** |
| --- | --- | --- | --- | --- |
| verbatimScientificName | Species | B-Taxon  I-Taxon | Original character string provided as species name or taxon identifier by the data provider. | “Pan paniscus”; “Arachnida”  “C. planirostris”  “L. brasiliense” |
| verbatimLocality | Loc | B-Loc,  I-Loc | The specific description of a place. | “Florida”; “Germany” |
|  | LocDate |  | The date at which a Species was observed in a specific location (LOC) | “10.09.2009”, “3 March 2022”, “24.IV.2009”.  “Type material: Holotype: Male collected from banana agro-ecosystems near Raver (21.25  ° N, 76.03° E), Distt. Jalgaon, Maharashtra, India, **21- 25 December 2012**, Seema Keswani,  deposited at Arachnology Museum, Forest Training Institute, Chikhaldara, Maharashtra-India” |
| verbatimCoordinateSystem | CoordSys | B-CoordSys | See: [https://dwc.tdwg.org/terms/#dwc:verbatimCoordinateSystem](https://dwc.tdwg.org/terms/#dwc:verbatimCoordinateSystem) | decimal degrees, degrees decimal minutes, degrees minutes seconds, UTM,  EPSG:4326, WGS84, NAD27, Campo Inchauspe, European 1950, Clarke 1866  [http://wiki.gis.com/wiki/index.php/Datum_(geodesy)#List_of_Datums](http://wiki.gis.com/wiki/index.php/Datum_(geodesy)#List_of_Datums) |
| verbatimCoordinates | Coord | B-Coord  I-Coord | See: [https://dwc.tdwg.org/terms/#dwc:verbatimCoordinates](https://dwc.tdwg.org/terms/#dwc:verbatimCoordinates)  Some papers will have coordinates provided by the authors with greater precision than checking a gazetteer for locations. Could be in several datums and coord systems. | (39.8210N 2.7951E),  (-41.0983, -121.1761),  17T 630084 4833438  41 05 54S 121 05 34W  -77.508333, 164.754167  77° 30.5' S, 164° 45.25' E 77° 30' 29.9988" S, 164° 45' 15.0012" E  -1314485.732632, 358267.239976 |
| verbatimTraitName | Trait | B-Trait,  I-Trait | Original character string provided as trait label by the data provider. This may include abbreviations of trait names. | “Tail length”, “greatest length of skull”, “GLS” |
| verbatimTraitValue | TraitVal | B-TrVal,  I-TrVal | Measurement trait values:  Trait Value can be a numerical measurement (56-57, 12).  Describing the ranges of calculated data including min, max value and number of specimens.  This is a very abstract form of an entity as measurement ranges can hold mean, SD, ranges (min, max) which cannot be separately tagged. So it should be tagged as a compound. | 33.57 (31.36-36.56, 50)  29.5-34.6  6.5, 7.5  12.7 ± 0.2 |
| verbatimTraitUnit | Unit | B-Unit | The unit value of the measured trait. These values should only be tagged if they are describing the unit of the trait value measurement. Irrelevant data units should be avoided. | g, mm, cm, kg, inches, in |
| individualCount | Count | B-Count | Number of specimens. It must be a numerical value either in number or textual form.  It should be only tagged as mentioned separately in examples. If they are part of the range then do not tag them separately as (23-25,**13**) this is part of the range now. | **15** males**,**  **One** female**, three** of the specimens.  **10** females  Specimen of **15** organisms,  n = **16** |
| StatisticalMethod | Stat | B-Stat,  I-Stat | For aggregated measures, the method for data aggregation or averaging as well as the variation or range. | means (mm; ranges)  SD (mm; range, coefficient of variation) |
| sex | Sex | B-Sex | The sex of the biological individual(s). This should imply sexes whose trait data is given. and can be either a noun or a gender symbol, ♂, ♀, ☿. | “male”, “female”, “unknown”, “hermaphrodite”, “2 **♂♂**”, “m”, “f”, “mf”, “mm”, “ff”, |
| lifeStage | LStage | B-LStage  I-Lstage | The age class or life stage of the biological individual(s). | “juvenile”, “juv.”, “j.”, “jj.”, “subadult”, “adult”, “ad.”, “egg”, “larva”, “pupa”, “spiderling”, “instar”, “nymph”. |
| measurementRemarks = citation in the text | Ref | B-Ref,  I-Ref | A citation that references the measurements of trait data. There can be many citations in the document. But only the ones that relate to providing trait information should be tagged. | **Gimenez and Giannini (2016)**  provided means (mm; ranges) of craniodental measurements.  **Peters et al. (2002)** reported the following means  (mm; ranges) for six females and 12 males, respectively.  In Brazil ( **Vizotto and Taddei 1976** ), means  SD (mm; range, coefficient of variation) for 15 males and 15  females, respectively |
| measurementDeterminedDate | Date | B-Date,  I-Date | The date on which the MeasurementOrFact was made. | “1900”, “April 1900”, “12 Feb 1900”, “12.02.1900”, “02-12-1900” |

**NOTE:** You will need this table to confirm whether the annotations are correctly done or not. These tags are saved in the INCEpTION project template which will be provided as a starter framework to annotate any document. By selecting the text and then selecting the above mention tag the annotation will be done. Some of the tags may be pre-annotated and you can scrutinise them by deleting and updating with the correct one, where required.

# 2. GUIDELINES FOR ANNOTATIONS

## 2.1 MATERIAL NEEDED

- **Computer.** Most OS’ should be fine. Needs to be able to run your choice of web browser.
- **External mouse.** One bug in INCEpTION can only be bypassed if you have an external mouse with a mouse wheel.

## 2.2 HOW TO ANNOTATE IN INCEPTION

1. **Locate relevant data**

Read the document. Identify only those sections where either trait measurement or occurrence of species are specified.

Measurements often included an animal’s phenotype (head circumference, body length, weight, etc) and their secondary information (e.g: life stage, count of Individuals for those measurements, etc).

**Example of trait measurement from data:**

In Brazil (Vizotto and Taddei 1976) , means + SD (mm; range, coefficient of variation) for 15 males and 15 females, respectively, were: head-body length, 55.7 + 0.5 (51.0-59.0 , 3.3) , 52.9 + 0.3 ( 50.5-54.5 , 2.4) ;

**Extracted labels of above mentioned traits as described in TABLE 1 ANNOTATION ENTITY TAGS AND DEFINITIONS.**

| **Text** | **Tag** |
| --- | --- |
| Vizotto and Taddei 1976 | Ref |
| means + SD | Stat |
| mm | Unit |
| range | Stat |
| coefficient of variation | Stat |
| 15 | Count |
| males | Sex |
| 15 | Count |
| females | Sex |
| head-body length | Trait |
| 55.7 + 0.5 | TraitVal |
| 51.0-59.0 | TraitVal |
| 3.3 | TraitVal |
| 52.9 + 0.3 | TraitVal |
| 50.5-54.5 | TraitVal |
| 2.4 | TraitVal |

1. **Annotate an entity**
2. Select the text to be annotated with mouse-1. Only select the relevant text. Do not include unnecessary blank or whitespace, parenthesis, commas unless they are part of a tag.
3. Keep the tag selected and then annotate the tag from the combo box **value** given on the right side.

Example:

Here in this example condylobasal length has been selected by the user. Only the tag name is highlighted, leaving the comma or any other space unselected. If the trait name is already highlighted then confirm that tag is correctly placed. If not in both cases switch to tag the specific genre.

Fig 1: Select Text


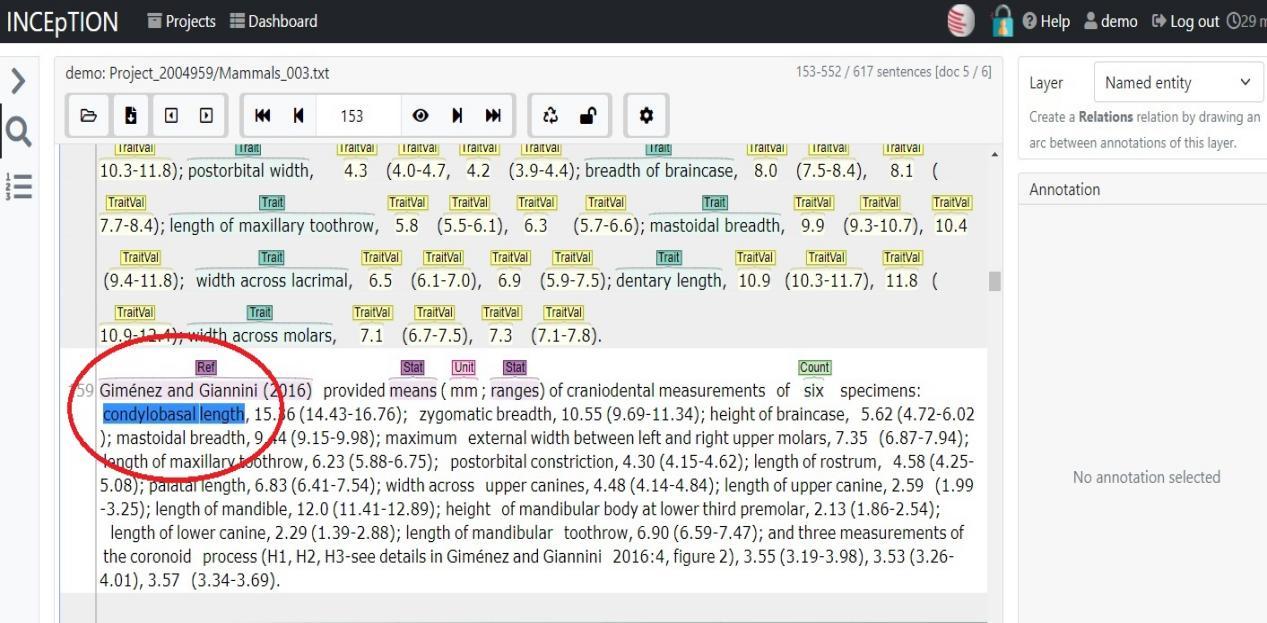


Fig 2: Select Text


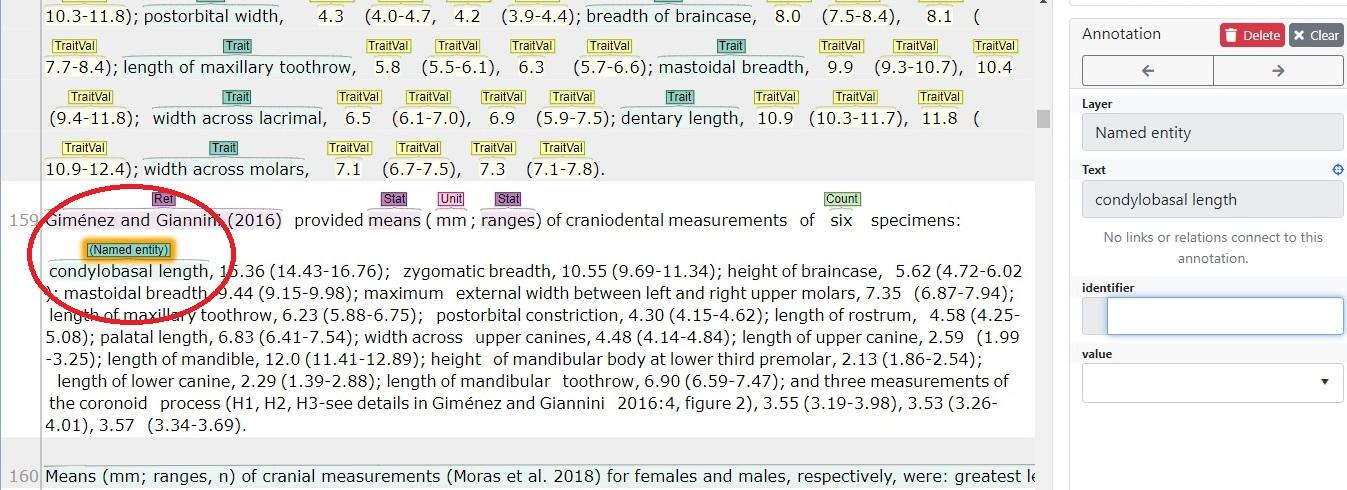


Fig 3: Tag the Entity


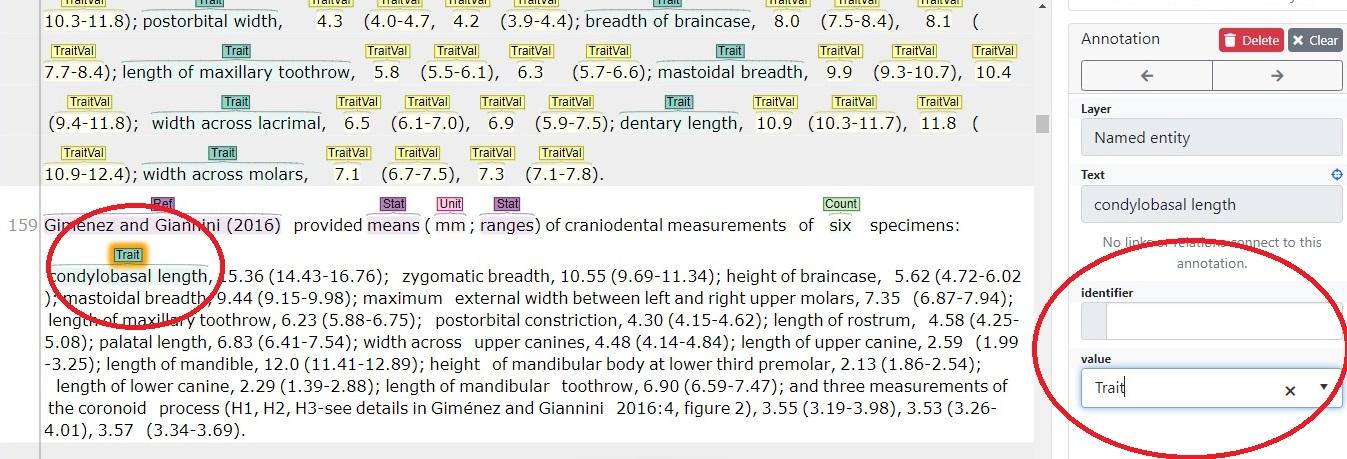


**NOTE:**

If you think you’ve made a mistake, you can delete any named entity or relation by selecting them and pressing **Delete.**

## 2.3 PREVIEW OF ANNOTATED DATA

An annotated measurement before and after annotation will look like this:

Ranges of skull measurements (mm; n) of specimens from Argentina (Barquez et al. 1999, 2011; Idoeta et al. 2012) were: greatest length of skull, 13.30-17.80 (13); condylobasal length, 14.78-16.74 (12); least interorbital breadth, 6.18-7.18 (13); zygomatic breadth, 10.56-11.58 (10); postorbital constriction, 3.91-4.60 (13); breadth of braincase, 7.68-8.30 (13); length of maxillary toothrow, 5.89-7.00 (13); palatal length, 6.16-7.10 (13); mastoidal breadth, 9.68-11.50 (10); length of mandibular toothrow, 6.30-7.50 (13); length of mandible, 11.69-13.58 (12); width across canines, 4.07-5.0 (13); width across molars 6.82-8.0 (13).

Fig4: Annotated in Inception


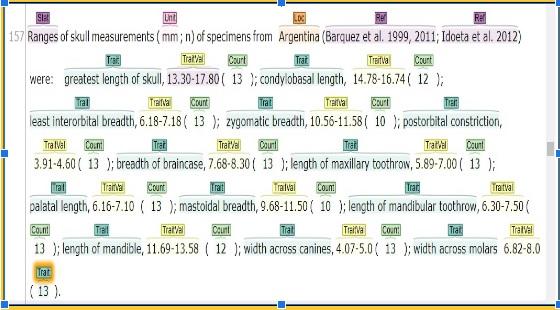


## 2.4 EXAMPLES & CAUTIONS FOR ANNOTATORS

There are some rules and norms that have to be followed while annotating each entity. This is to make sure they are consistent for the follow-up analysis. There’s quite a few but they’re not complex. Repetition while handling multiple scenarios and data variances in publication formats will see you quickly become familiar with them.. Here’s a few of them as per each tag:

### 2.4.1 Stat (Statistical Method)

Stat measures indicate the numerical or statistical method of measurement. This may include ranges, means with SD (Standard Deviation), Coefficient of variation etc. Each statistical method should be independently tagged as a ‘Stat’ method, only if there is a resultant value present.

***Example 1.* Ranges:** A range is the stat measure which is related to the range of any trait value. In the example below: only the ranges of the trait values are provided. The range values can be found later as 54-63, 24-30 etc. So the statistical measure as **ranges** is tagged.


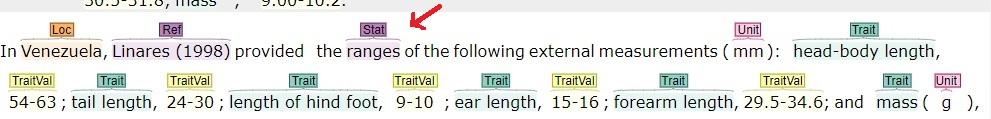
Fig5: Range as Stat

***Example 2.* Means and Range:** In this example two statistical measures are provided with relevant data i-e **means** and **range**. Therefore they are separately tagged. Later their corresponding trait values will also be separately tagged.

**Fig6: Means and Range as Stat**


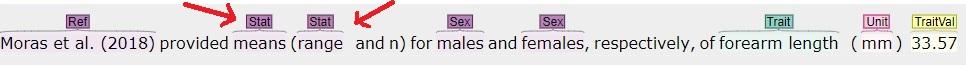


***Example 3.* Means+-SD, range and Coefficient of Variation:** In this example three statistical measures can be identified.) **means** is **coupled with SD** and is taken as one measurement. **Range** and **coefficient of variation** are other two stat measure tags.

**Fig7: Means +-SD,range and Coefficient of Variation as Stat**


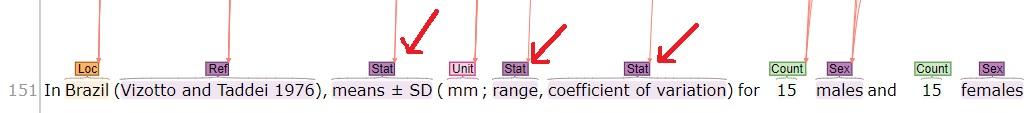


***Example 4.* means+-SD, range:** In this example two stat measures means+-SD and range are separately tagged.

**Fig8: Means +-SD,range as Stat**


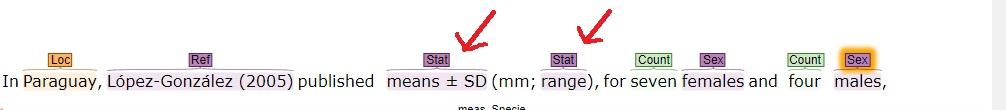


### 2.4.2 Species (verbatimScientificName)

A species’ scientific name will almost always be written in two specific ways: as a binomial name of genus and specific name (e.g. *Macrothele calpeiana*) or as a binomial of genus abbreviation and specific name (*M. calpeiana*), the latter being used only after the non-abbreviated binomial has already been used in the paper. In some taxa some species might go further and have a sub-species name after the binomial (e.g: *Panthera tigris sumatrae, P. tigris tigris*).

Example

**Fig9 : Species Tag**


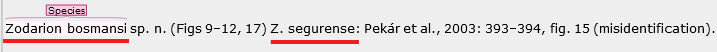


### 2.4.3 Trait and TraitVal (verbatimTraitName & verbatimTraitVal)

The trait names and their values are the most important named entities to annotate and as such if you have annotated a trait value you MUST annotate a species name and a trait name. Secondly, if a TraitName is not explicit in text - for example, if you have a measurement of a tail but what measurement it is (i.e: circumference, length, etc.) is not specified - then you must annotate it anyway to be interpreted in posteriority.

Example

The Trait Names tagged as **Trait** should have their respective TraitValues as TraitVal in the measurement. Consider this example, where the trait names include a whole expression including “length of”, “breadth of”, “height of”, etc. These components are essential information comprising the traitname.

**Fig10: Trait as Tag**
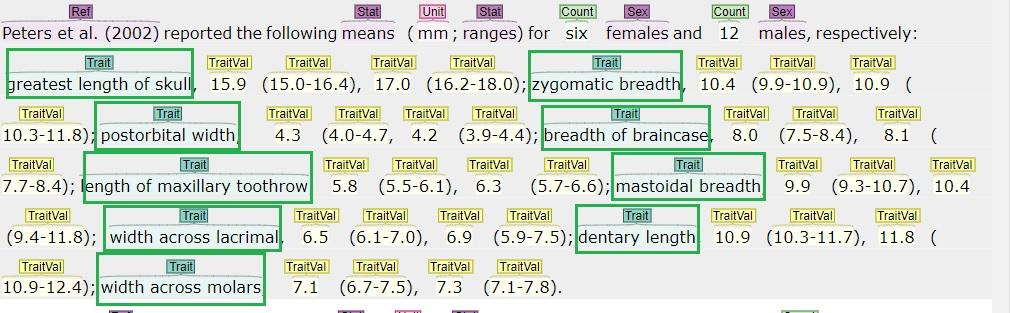


The trait names in the above fig are boxed green. You can see that the whole trait with its measured terms are included for tag.

***Example 1.*** Next we are interested in Trait Values which could be measured by stated stat values as means, ranges etc. In this example the Trait Values are based on Stat values means and ranges already mentioned as Stat tags. The Trait Values are tagged accordingly. Additionally, consider that occasionally the trait name might be separated. In these situations annotate the rightmost term, to be interpreted in posteriority.

Fig11: TraitVal as Tag


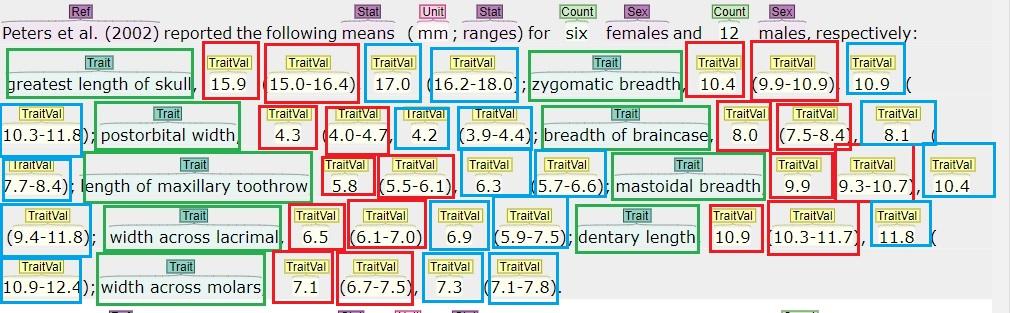


***Example 2.*** In this example, the authors have chosen to organise their measurements by body segment, creating sentences starting with a segment like “prosoma” and then specifying the measurement. Both “long” and “wide” refer to a measurement of the prosoma and could be rewritten as “length of prosoma” and “width of prosoma”, for example.

Fig12: TraitVal as Tag


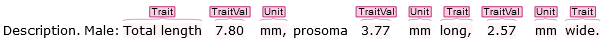


### 2.4.4 Ref (References)

When annotating, do not include parenthesis in the tagging except if they’re part of the reference’s year. E.g: tagging an in-text citation as “Author (2020)” is correct, tagging “(Author A 2020, Author B 2021, Author C 2022)” as three separate instances of “(Author A 2020” , “Author B 2021” and “Author C 2022)” is incorrect as they should be annotated as “Author A 2020”, “Author B 2021” and “Author C 2022”.

### 2.4.5 Loc (LocationofTaxon)

Sometimes a trait value or species is attributed to a location that is in fact a continuous string of location organisational units, such as “Portugal, Lisbon, Campo Grande”, which are country, district and parish, respectively. In these situations the entire string should be annotated as a single location.

Fig13: Loc as Tag


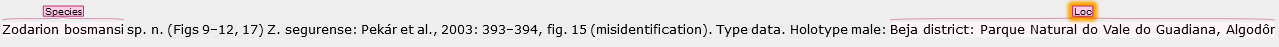


### 2.4.6 Count (individualCount)

***Example 1.*** Counts are the integer numbers of individuals associated with a given value **only** and do not include the common mathematical notation of “n =” denoting sample size.

Fig14: Count as Tag


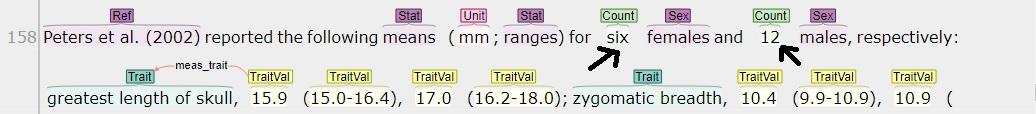


# 3. RELATIONS ANNOTATION GUIDELINES

Relations establish how the named entities relate to each other. Most are made particularly via TraitValue. A trait value is the core measurement value that is linked to the rest of its dependent attributes. A trait value describes one measurement with a complete set of values linked through it. Relations are annotated when a complete annotation process is done on the document or after a particular set of annotations are completed that can constitute relations. However, you should always consult Tables 2 and 3 at the **end of this section**.

When you create a relation, select the annotated term. Keep it selected. Drag the mouse button, an arc will emerge. Drop the arc to the entity you want to link with, e.g Trait. **WARNING**: in the current version of INCEpTION there is a graphical bug that may stop you from creating relations between two terms very distant between each other in the text. The “arrow” it creates will disappear but if you scroll down you’ll still be able to create the relation. However, this only works with external mice, it will not work with laptop trackpads!

Fig 15 : Create Relation between TraitVal and Trait.


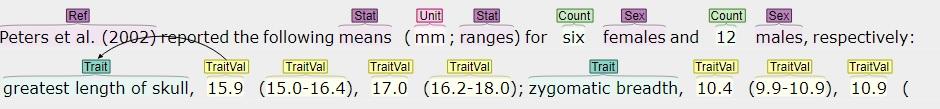


Once the link is established, on the right side the information will be displayed. Confirm the relations are correctly done. **From** and **To** tabs will show the values i-e TraitVal and Trait in this case. From the meas_prop drop down select the appropriate relation meas_trait in this case. As TraitVal is linked to Trait its meas_trait is selected.

Fig 16 : Relation Values between TraitVal and Trait.


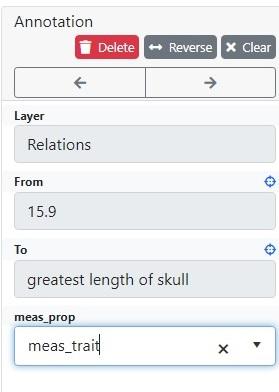


The relation **meas_trait** will be shown between the entities.

Fig 17 : Relation between TraitVal and Trait.


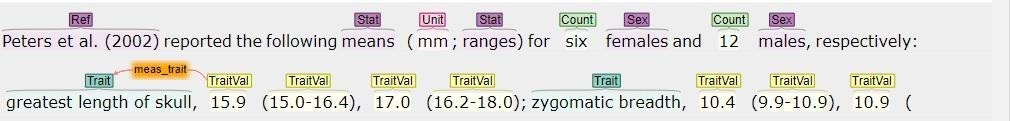


Likewise, create relations between one particular TraitVal with all its value. In Fig15 TraitVal (15.9) has to be linked with its attributes :

| Trait | **greatest length of skull** |
| --- | --- |
| Sex | **females** |
| Count | **six** |
| Stat | **means** |
| Unit | **mm** |
| Ref | **Peters et al. (2002)** |

Each Traitval should be linked with every bit of information that makes its measurement complete. There could be repetitive links to the same entities for such.

## 3.1 Caution for Relations Making

Once the annotation process is complete, please save it in the webanno.tsv format. You can upload the file later as well in case you think you’ve forgotten something. Regardless, if you’re using the online browser version of INCEpTION any changes you make should be automatically saved. The status of the annotations can be restored if any problem arises.

### Relations

All relations are based on TraitVal. The source or the base of all relations is TraitVal. With a trait value you have to link all the rest of corresponding tags.

### Occurences

A bit trickier but not by much. The starting term is Species with only one exception, GIVEN COORDS:


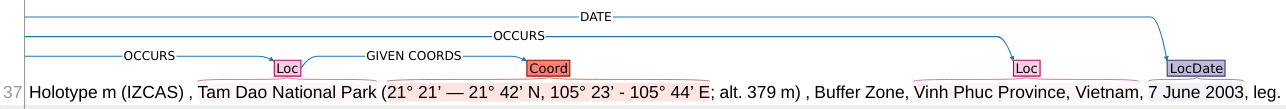


TABLE 2: RELATION TABLE BETWEEN ENTITIES. TRAITS

| **NAME** | **ENTITY 1** | **ENTITY 2** | **RELATION** | **REMARKS** |
| --- | --- | --- | --- | --- |
| **MEASUREMENT RELATIONS** | | | | |
| **meas_Species** | TraitVal | Species | **verbatimTraitValue**–**verbatimScientificName** | Trait Value of Species linked with Species Name |
| **meas_trait** | TraitVal | Trait | **verbatimTraitValue**–**verbatimTraitName** | Trait Value linked with Trait Name |
| **meas_Unit** | TraitVal | Unit | **verbatimTraitValue**–**verbatimTraitUnit** | Trait Value linked with its Trait Unit |
| **meas_Stat** | TraitVal | Stat | **verbatimTraitValue**–**StatisticalMethod** | Trait Value linked with Statistical Methods (e.g range, means+-SD, SD, coefficient of variations) |
| **meas_Loc** | TraitVal | Loc | **verbatimTraitValue**–**verbatimLocality** | The Location where the measurements were made is linked with TraitValue. |
| **meas_Count** | TraitVal | Count | **verbatimTraitValue**–**individualCount** |  |
| **meas_Sex** | TraitVal | Sex | **verbatimTraitValue**–**Sex** |  |
| **meas_LStage** | TraitVal | LStage | **verbatimTraitValue**–**LifeStage** |  |
| **meas_Ref** | TraitVal | Ref | **verbatimTraitValue**–**References** |  |
| **meas_Date** | TraitVal | Date | **verbatimTraitValue–measurementDeterminedDate** |  |

TABLE 3: RELATION TABLE BETWEEN ENTITIES. OCCURENCES.

| **NAME** | **ENTITY 1** | **ENTITY 2** | **RELATION** | **REMARKS** |
| --- | --- | --- | --- | --- |
| **SPECIES LOCATION RELATION** | | | | |
| **OCCURS** | Species | Loc / Coord | Species→Loc  (One-Many) | Occurrence of a species at any location. If unpaired, i.e.: is described only with a set of coordinates, Coord is the end term, otherwise Loc takes precedence. |
| **DATE** | Species | LocDate | Species→LocDate  (One-Many) |  |
| **GIVEN COORDS** | Loc | Coord | Loc→Coord  (One-One) | If a location is described with both a Loc and Coord entities, this relation is established between them. |

# 4. End notes

If you can think of anything specific please suggest it, but we think this document should have everything important. The relations are a last easy step, the most challenging part are the entities. If you have any questions, please reach me at vasco.branco@helsinki.fi.
